# Creating an online educational intervention to improve knowledge about systematic reviews among healthcare workers: mixed-methods pilot study

**DOI:** 10.1186/s12909-022-03763-3

**Published:** 2022-10-14

**Authors:** Marina Krnic Martinic, Snjezana Malisa, Diana Aranza, Marta Civljak, Ana Marušić, Damir Sapunar, Tina Poklepovic Pericic, Ivan Buljan, Ruzica Tokalic, Dalibor Cavic, Livia Puljak

**Affiliations:** 1 Department of Otorhinolaryngology, University Hospital Split, Split, Croatia; 2 Center for Evidence-Based Medicine and Health Care, Catholic University of Croatia, Ilica 242, 10000 Zagreb, Croatia; 3 University Department of Health Studies, University of Split, Split, Croatia; 4 Department of Research in Biomedicine and Health, University of Split School of Medicine, Split, Croatia; 5 Department of Histology and Embryology, University of Split School of Medicine, Split, Croatia

## Abstract

**Background:**

Lack of knowledge about systematic reviews (SRs) could prevent individual healthcare workers (HCWs) from using SRs as a source of information in their clinical practice or discourage them from participating in such research. In this study, we aimed to explore in-depth the opinion of a sample of HCWs about the newly created online educational intervention designed to improve knowledge about SRs.

**Methods:**

We created a brief online educational intervention on SRs, consisting of 11 textual modules. We evaluated it among practicing HCWs who graduated from a university-level health sciences program using a mixed-methods pilot study that consisted of pre- and post-intervention questionnaires and qualitative evaluation via semi-structured interviews. We assessed participants’ knowledge about SR methodology before and after the intervention, and compared the responses. We sought their opinions about the characteristics of SRs. Also, the participants were presented with four scientific abstracts, where they were asked to distinguish whether those abstracts presented summaries of a systematic or a non-systematic review.

**Results:**

Twelve participants took part in the study. In the pilot study, the participants’ knowledge about SRs was improved after the intervention compared to the baseline. Participants provided positive feedback regarding the educational intervention. Suggestions to improve the educational intervention were to provide more details about the forest plot, add more digital content or images, provide more details about the methodological steps of an SR, add descriptions about practical applications of SRs and provide links to additional educational materials. The participants suggested that HCWs could be motivated to take part in such an education if it is offered as continuing medical education (CME) course or credit for academic/career advancement.

**Conclusion:**

HCWs provided positive feedback about the newly designed online educational intervention on SRs; they considered it an appropriate tool for learning about SRs and resulted in increased knowledge about SRs. In addition, participants gave suggestions for improving education, which can be used to tailor the education for end-users. In future studies, it would be useful to examine the effectiveness of the modified educational intervention on increasing knowledge in a larger sample and in the form of a randomized controlled trial.

**Supplementary Information:**

The online version contains supplementary material available at 10.1186/s12909-022-03763-3.

## Background

Online educational interventions are easy to implement, low-cost, and can be easily refined and stored for later use if needed. A number of studies can be found in the literature which evaluated educational interventions conducted via the Internet related to various topics in the field of medicine [1, 2]. Online evidence-based medicine (EBM) education could improve clinicians’ skills in EBM, particularly when it is conducted during vocational training [3]. Such educational interventions can also be used to teach targeted individuals about the best evidence available to inform medical practice. The implementation of best evidence in decision-making regarding health is interchangeably called EBM, evidence-based practice (EBP) and evidence-based healthcare (EBHC) [4].

A systematic review about physicians’ knowledge, attitudes and practice toward evidence-based medicine concluded that they have a generally positive attitude toward EBM, and most of them believe that its implementation improves patient care. However, their self-reported awareness and knowledge regarding EBM concepts and the relevant databases are generally poor [5]. As reported in the literature, both individual factors (such as personal interest) and organizational factors (workload, hospital requirement) have an effect on physicians’ attitudes and their EBM/EBP skills [6]. Poor knowledge about SRs among individuals in charge of European PhD programs was demonstrated in a study conducted by Puljak and Sapunar [7].

Several studies have examined the effect of online educational interventions among healthcare workers (HCWs) on EBP knowledge and have shown that such interventions are effective [8–10].

In 2007, Shuval et al. published the results of a controlled trial examining the effect of an educational intervention on EBM among family physicians in Israel [10]. The trial showed that educational intervention had significantly improved the level of knowledge and attitudes about EBM. On the other hand, the educational intervention did not significantly impact the clinical practice of physicians in terms of prescribing medications and prescribing tests [10].

Another study evaluated the effectiveness of an accelerated education program on EBP and the application of EBP among nurses employed in acute care facilities [8]. The study showed that an accelerated, eight-week educational program influenced a statistically significant positive change in nurses’ beliefs and attitudes about EBP [8].

A study aimed to describe and evaluate the acceptability, utility, satisfaction and applicability of the general practitioner registrars’ experience with the online course demonstrated generally positive reactions towards the course and the concept of EBM, stating that the course improved their confidence, knowledge, and skills and consequently impacted their practice [3].

However, we are not aware of educational interventions specifically devoted to systematic reviews (SR) and their methodology. Potentially, the lack of knowledge about SRs could prevent individual HCWs from using them as a source of information in their clinical practice or discourage them from participating in such research. Educational interventions about SR methodology could potentially increase the knowledge of HCWs about SRs and encourage their use in solving clinical problems.

Before using such educational interventions on a large scale, it is important to test them among HCWs to obtain their feedback and potentially improve the intervention. In this mixed-methods pilot study, we aimed to conduct preliminary testing of our intervention and collect in-depth opinions of a sample of HCWs about the newly created online educational intervention designed to improve knowledge about SRs, as well as about the usefulness, applicability and experience with the course.

## Methods

### Study design and reporting

This was a mixed-methods pilot study, which included testing of the newly designed brief online educational intervention with pre- and post-intervention questionnaires and qualitative evaluation via semi-structured interviews. For reporting, we used Consolidated Criteria for Reporting Qualitative Studies (COREQ) [11].

### Ethics

The study protocol was approved by the Ethics Committee of the Catholic University of Croatia (Approval: Class 641-03/21 − 01/03; Urbroj: 498-03-02-06-02/1-21-02). All participants provided written informed consent. All methods were performed in accordance with the relevant guidelines and regulations, including the Declaration of Helsinki.

### Protocol registration

The study protocol was published on Open Science Framework (OSF) platform (available at https://osf.io/pj79b/). There were no deviations from the protocol.

### Study participants and sampling

Participants were practicing HCWs with completed university-level health sciences studies in Croatia. Participants were collected by purposeful sampling among alumni of the graduates of university-level health sciences studies in Croatia. Participants were personally contacted based on the recommendation of their former teachers. The number of participants who refused to participate in the survey and their reasons were documented.

Individuals nominated by their teachers were sent an e-mail invitation for participation in the study, followed by a total of three reminders 4 days apart. Text of invitation is provided in Supplementary file 1. If they accepted the invitation, an appointment was made to participate in the educational intervention and follow-up interview. They received an e-mail link for the interface hosting the educational intervention at the agreed time. In the first part of the appointment, participants accessed the online interface containing the questionnaires, including the pre-intervention and post-intervention questionnaire (Supplementary file 2), and the educational intervention on SRs (Supplementary file 3).

### Development of intervention

The intervention was developed by the study authors, experienced medical and health sciences educators and methodologists. The text of the intervention was based on Cochrane’s online learning modules “Cochrane Interactive Learning: Conducting an Intervention Review” [12]. The text of the intervention was organized into 11 short text modules, briefly describing the basic concepts of an SR (Supplementary file 3). The modules were provided uninterrupted, sequentially, from the first to the last one. The estimated reading time required for the completion of each module was 2 min.

Learning objectives of the intervention were the following: to be able to define EBM, identify different levels of evidence, recognize an SR, describe the process of defining a clinical question, describe the steps for writing an SR protocol and registering it, to define literature search and screening, to recognize the risk of bias assessment, to recognize the process of data analysis and interpretation in SRs. In addition to acquiring knowledge, the learning objective was also to apply the knowledge by differentiating an abstract presenting an SR and a non-systematic narrative review.

The text of the intervention was revised iteratively, based on the suggestions from the team. The intervention was designed as an asynchronous online education without live education or interaction. To motivate HCWs to participate in the study, we offered them certificates of attendance issued by Cochrane Croatia.

### Online questionnaires

The aim of the questionnaire was to study sociodemographic data, knowledge of SRs and participants’ opinions about the SR characteristics.

The questionnaires consisted of multiple parts. We did not include any assessments between the modules. The post-intervention knowledge assessment and questions regarding abstracts were shown only after all the modules were presented. The initial segment included general questions addressing the sociodemographic characteristics and participants’ familiarity with SRs (Supplementary file 2). Next, there were nine pre-intervention questions assessing knowledge about the SR methodology. They were followed by six statements about the characteristics of SR methodology on which the participants were asked to express their agreement via a Likert scale ranging from 1 (completely disagree) to 5 (completely agree) (Supplementary file 2). The six characteristics of SRs that the participants were given were not a part of the knowledge test. Those six characteristics were proposed as the defining characteristics of SRs in our previous study [13].

For example, one of the items assessing knowledge was “Systematic reviews must contain a meta-analysis”; the correct answer to this is “No”, as an SR may or may not contain a meta-analysis and sometimes meta-analysis is not done due to clinical or statistical heterogeneity.

After that, the participants proceeded to the educational materials divided into 11 methodological units (Supplementary file 3).

After the educational intervention, the participants were asked to answer post-intervention knowledge assessment questions, which were the same nine questions as asked in the pre-intervention questionnaire. Then, the participants needed to assess the six statements about the SR characteristics again. In the last section of the questionnaire, the participants were presented with four different research abstracts from scholarly journals, two abstracts describing SRs [14, 15] and two abstracts presenting non-systematic narrative reviews [16, 17] (Supplementary file 4). The participants were asked to try to assess which abstract reported an SR and which did not.

### Qualitative evaluation

*Team and reflexivity*

All interviews were conducted by one investigator, a female (MKM) medical doctor, an otorhinolaryngology specialist who had previous experience with a qualitative study in the form of an interview [18].

The participants did not know the investigator who conducted the interviews. This study is thematically associated with the two studies formerly done by the investigator MKM [13, 18]. The first study explored the attitudes of editors of core clinical journals about the originality of SR and concluded that although the majority of editors considered SRs original research projects, the concept of originality of a study is still elusive and evolving [18]. The other study searched for a standardized, uniform and unambiguous definition of a SR and concluded that such a definition of a SR still does not exist in the literature [13].

*Methodological orientation and theory*

We used a qualitative description (QD), a method relevant in research projects aiming to gain firsthand knowledge of participants’ experiences with a particular topic. With QD, in the analytical process and presentation of data, researchers stay closer to the data, presenting pure description [19].

*Conduct of interviews*

Immediately after completing the educational intervention and completing the questionnaire in the online interface, an interview about the educational intervention was conducted via the Zoom platform. All interviews were conducted by one researcher (MKM). Participants gave informed written consent to participate in the study (Supplementary file 5). The interviews were conducted individually. Participants could choose to participate in the interview through various platforms, including Skype, Zoom, MS Teams, or some other platform of choice. During interview, participants were asked pre-defined questions that were defined in the study protocol, and that are listed in Supplementary file 6. An audio recording was made for each interview. The duration of each conversation was recorded. All recordings were saved on a secure server.

*Interview transcripts*

One author (MKM) transcribed all the interviews, and another author (SM) checked the transcripts. The interviews were not repeated. The transcripts were sent to the participants for insight and approval.

### Analysis and results

For analysis, the names of all participants were coded. Transcripts were analyzed through QD, aiming to present a rich, straight description of an experience [19]. One author (MMK) coded the text, and another team member (SM) checked the coding; all disagreements were resolved with the help of a third author / evaluator. The codes were entered into an MS Excel (Microsoft Corp., Redmond, WA, USA) spreadsheet for further qualitative analysis. Entire sentences were transcribed from the original transcripts of the interviews in order to present the original thoughts of the participants. The quotations were listed under the ordinal number of the participants. Participants were not asked for feedback on the results.

The results of categorical variables from the pre- and post-intervention knowledge assessment were expressed as frequencies and percentages. Results for continuous data were expressed as medians and interquartile ranges (IQRs). For data analysis, we used GraphPad Prism 6.0 (SPSS Inc., Chicago, IL, USA).

## Results

Twenty-two individuals were invited to participate in the study. After sending the first official invitation to participate in the research, two invited individuals declared they were unable to participate, one for personal reasons and the other due to workload. Eight individuals did not respond to the e-mail invitation even after three reminders.

Twelve individuals participated in the study. The first interview was conducted on April 7, 2021, and the last on April 27, 2021.

### Participants’ characteristics

Participants’ characteristics are shown in Table 1. Most participants were women, and completed the nursing study program. All participants were employed at the time of the interview. Most were employed as HCW. Eight participants who worked as HCWs had a range of 5 to 29 years of experience as HCWs, with an average length of working in healthcare of 18 years. The age of the participants ranged from 30 to 53 years, with an average age of 39 years. We did not observe any differences between women and men or different roles in their responses.

**Table 1:** Participants’ characteristics

Variable	Response	N
Sex	Woman	9
	Man	3
University completed	Catholic University of Croatia	5
	University Department of Health Studies, University of Split	5
	Medical Faculty of the University of Zagreb	2
Study program completed	Nursing	11
	Radiology technology	1
Employed	Yes	12
Employed as a healthcare worker	Yes	8
	No	4
Median knowledge score* before educational intervention, IQR	3 (IQR 3-3.5)	
Did you hear about systematic reviews as a type of research before this study?	Yes	12
Where did you hear about systematic reviews?	University	12
How many systematic reviews did you read before this study?	At least one	11
	None	1
Did you ever participate in producing a systematic review?	Yes	1
	No	11

Acronym: IQR = interquartile range

*Participants rated their knowledge of SRs on a scale of 1 (insufficient knowledge) to 5 (excellent knowledge)

### Pilot evaluation of the intervention via questionnaires

Detailed results with tables of the pilot evaluation of the intervention via pre-intervention and post-intervention questionnaires are available in Supplementary file 7. All raw data collected within the pre-intervention and post-intervention questionnaire are available in Supplementary file 8.

### Knowledge assessment

All participants had more correct answers on the post-intervention knowledge test compared to the pre-intervention (Table 2). Both before and after the intervention, most of the wrong answers were recorded for the two items about the graphs used in SRs – funnel plot and forest plot (Supplementary file 7). Overall, the participants had 20% more correct answers after the intervention than before the intervention.

**Table 2:** Difference in knowledge before and after the educational intervention

Variable	Before intervention	After intervention
Participants providing all correct answers to questions about SR	0	4
The median number of correct answers to questions about SR	5.5 (IQR 5–7)	7 (IQR 6.5-9)
Overall possible correct answers to questions about SR	67/108 (62%)	89/108 (82%)
Participants’ consideration of the proposed characteristics of SRs as necessary	82% (combining all the participants’ answers, 59 of 72 SRs characteristics were considered necessary)	99% (combining all the participants’ answers, 71 of 72 SRs characteristics were considered necessary)

### Participants’consideration of proposed characteristics of systematic reviews as necessary

Participants’ consideration of the proposed characteristics of SRs as necessary before the intervention are shown in Supplementary file 7. Most participants fully agreed with all the claims about SRs; the percentage of complete agreement was 82% (59 participants completely agreed with 72 statements) (Supplementary file 7).

After the intervention, all participants completely agreed with all six proposed SR characteristics in the post-intervention questionnaire, except for the first statement (a research question is defined) on which one participant did not produce an answer. The number of participants who fully agreed with the statements about the characteristics of SRs after the education was 71/72 (99%), representing an increase of 17% compared to the pre-intervention questionnaire (Supplementary file 7).

### Using systematic reviews in practice after the education

As part of the post-intervention questionnaire in the SurveyMonkey platform, participants were asked to indicate how they would use SRs in their clinical practice. Eight participants (67%) cited specific applications such as resolving doubts about the treatment choice, for example, regarding pressure ulcer care, postoperative care of ophthalmic patients, the comparison of radiological techniques or the question of postoperative analgesic effect in adult women or in the daily search for answers to clinical questions. One participant stated that they would use an SR to make a meta-analysis. One would use an SR in terms of reviewing patients’ medical records, and one participant stated that “*systematic reviews of literature can be used as a basis for developing standards for certain procedures / interventions, etc.*“.

One participant stated that they do not engage in clinical practice but use SRs to educate students.

### Finding answers to clinical questions

In the post-intervention questionnaire, participants were asked where they would look for an answer to a clinical question. Eight participants (67%) stated that they would look for an answer to a clinical question in the scientific literature, three (25%) would look for an answer in an SR of the literature, one would look for an answer in an Internet search engine such as Google. One participant would consult scientific literature, books and an Internet search engine. Finally, one participant would consult superiors in addition to the scientific literature.

### Correctly identifying abstracts of systematic reviews versus non-systematic narrative reviews

Finally, at the end of the interface, participants were presented with four abstracts (Supplementary file 4), two of which were abstracts of SRs and two abstracts of non-systematic narrative literature reviews. Eight (67%) participants correctly recognized the first abstract of an SR; 11 (92%) participants correctly identified the second abstract of an SR as such. Ten (83%) participants correctly recognized the first abstract, and 8 (67%) correctly identified the second abstract of the non-systematic narrative review.

### Qualitative evaluation

After going through the education and answering questions in the SurveyMonkey interface, the participants took part in a semi-structured interview. The duration of the interviews ranged between 5:00 and 16:41 min, with a median duration of 9:55 min (IQR 7:36 − 12:07 min).

The participants stated that they needed between 20 and 50 min to go through the entire interface with the educational intervention and questionnaires, with a median time of 32:30 min (IQR 28:45 − 36:15 min).

The following topics were addressed during the interview: the effect of the educational intervention on participant’s knowledge, appropriateness of the online format for educational intervention, appropriateness of duration of the education, content of the educational intervention and suggestions for improvement, motivating HCWs to participate in the education and the effect of the education on the use of systematic reviews in clinical practice.

### The effect of the educational intervention on participant’s knowledge

All 12 participants answered that the information received in the education changed their knowledge of SRs. Seven of them indicated that the education renewed or expanded their previous knowledge.

I2: „… *more expanded, because there were things I simply did not know, simply forgot because I don’t use it frequently… “*.

I4: *„…more of renewed the knowledge because I forgot it, I’ve learned about it, but I forgot it… “*.

One participant stated that they previously did not know that SRs evaluated biases, and one participant found that the described graphs were new to them.

Seven participants stated that, of all parts of the intervention, information given in the educational materials improved their knowledge about SRs the most.

I10: „*How to search the literature, what a systematic review really is, what needs to be done to satisfy systematic review criteria*…*”*.

Four participants declared that they benefited the most from the abstracts they were supposed to read, and two participants explained that they gained the most knowledge from the pre-intervention and post-intervention questionnaires. One participant stated all parts of the education were equally important for improving knowledge.

To the question if the participants had any dilemmas about SRs after finishing the education, eight participants answered they had no dilemmas, two participants stated they had additional questions about SRs, one participant was not sure if he had dilemmas or not, and one participant stated.

I8: „*I think the dilemmas have only just opened up.“*.

Despite the majority stating that they had no dilemmas regarding SRs, most participants had additional questions about SRs. One participant stated that they would like clarifications about the forest-plot graph, one participant stated that they would like clarifications about the meta-analysis, one participant stated that they had dilemmas about the hierarchy of evidence in medicine, and four participants stated they were unsure in deciding which of the abstracts they were supposed to read were SR summaries and which were not. One participant wondered whether there is a minimum amount of evidence that needs to be included in an SR in order for it to truly be an SR.

I12: „*What to me now is, let’s say, the question, what exactly is the minimum amount of evidence actually needed to be included in order for it to be called a systematic review? Is there any minimum number of studies that must be included, because I see later in those examples that one, two, several studies are included, so it occurred to me whether these are systematic reviews at all…* “?

### Online format is an appropriate way of intervention delivery

Ten out of twelve participants stated that this kind of online education is an appropriate method of learning about SRs, one that it is not an appropriate method, and one that it is a relatively appropriate method. Two of the participants who considered this kind of education appropriate for learning about SRs declared that during their formal education, they did not encounter any education specifically about SRs, or it was insufficient, so they considered this sort of education useful. Two participants stated that such online educations are appropriate because they provide access to education in time and conditions that depend exclusively on the user.

I9: *„…you have enough time to read 2–3 times on your own something that is less clear to you, which is different from the lecture, when the lecturer says it once and that’s it, this way you can read it several times, eventually write down something that is less clear to you and find about that information further…*“.

I4: „… *because I was just focused on reading, on reading and thinking about it and I just focused on it and I gave myself time*… “.

One of the participants who answered that this kind of education is relatively appropriate for learning about SRs said that it is still better when a teacher explains the lesson and gives additional examples.

One of the participants who answered that this sort of education is not appropriate for learning about SRs suggested that the education should be supplemented by an additional presentation of articles.

Ten participants stated that the online format is appropriate for the implementation of such an intervention among students and HCWs. Two participants stated that the online format is not suitable for implementing such an intervention. Nevertheless, four participants stated that they are proponents of learning face-to-face. One participant pointed out that the online format is particularly convenient during the pandemic:

I11: „*For me personally, it’s great, because anyway, you can slowly read something on your own and it’s easier somehow this way and especially now in this time of the pandemic when it may be harder to go somewhere to learn something, in fact, this is a very good way to learn, practice, repeat…“*.

One participant highlighted the advantage of online learning due to the possibility of independent organization of free time and time for learning:

I9: „*We are aware that when there are lectures, most people avoid it, and at home, we all still have some time to, say, do some education and work on ourselves, simply, it is not exactly a specific time when you have to be at a lecture, but in your own time you can always access education which is great…“*.

### The duration of the education was found appropriate

All participants agreed that the duration of online education was appropriate. Six participants stated that the duration was completely adequate or that they would not mind if the education was longer, two participants pointed out that if the education was slightly longer, it would probably be tedious, and one participant pointed out that the advantage of this education was that there was no time limit.

I12: „… *so enough, yes, I mean, if someone needs a little more time you can always take a little more time, there was no time limit*. “.

When asked what the optimal duration of online education would be, various proposals were given. The participants stated the duration of education from 15 min up to 60 min would be optimal. Nine participants agreed that the optimal duration of online education is 30–45 min.

### Content of the educational intervention would benefit from additional clarifications

All 12 participants agreed that the amount of text in the educational materials was appropriate. Participants generally stated that they received sufficient information about SRs with an acceptable amount of text.

I10: „*There wasn’t a lot of text, and yet everything that was supposed to be was presented.*“.

Also, all 12 participants stated that the content of the educational materials was written appropriately to online learning about SRs.

I8: „… *absolutely, it was understandable enough, even for some things I hadn’t heard so far, it was clear to me what they actually were*.“.

Participants were asked what their suggestions were for improving this education about SRs. Three participants stated that they would benefit from an additional explanation of the forest plot, three participants added that certain digital content or images could be added to the education. Two participants suggested that it would be better to conduct the same education face-to-face. One participant pointed out that it was unclear what steps needed to be done in an SR after screening for abstracts. One participant stated that additional emphasis could be placed on the practical application of education and additional explanations for recognizing an SR. One participant suggested adding links for additional educational materials.

### Motivating healthcare workers to participate in the education

When asked how we could encourage HCWs to participate in such educational programs, five participants stated that HCWs could be motivated by continuing medical education (CME) credits of professional chambers. Two participants suggested integrating the course in the formal education of HCWs as one of the tasks students need to complete, or as the criterion for career advancement in the education system.

I8: „ *I think that the only way health professionals can be encouraged to participate in any education is in the way that they benefit from it. Specifically, the course could be registered with the Croatian Chamber of Nurses, so the participants get some CME credits for that. Or let’s say, as I work in the school system, I need certain types of education for career advancements. If you create such courses for nurses who are employed in medical schools and make a series of 5 courses lasting an hour, issue a certificate for 5 h, it could be scored as one point for career advancement. I think that would motivate all my colleagues to attend.* “.

Five participants indicated that participation in such educational programs would depend solely on their own will and motivation for gaining knowledge and learning.

I2: „ *As a rule, there are always people, those who want to work and those who do not want to work, and those who do not want to work, you can encourage them all you want, without effect.*“.

Two participants pointed out that HCWs with a higher level of formal education will be more motivated to attend such a course than those with a lower level of schooling.

Two participants stated that HCWs could be encouraged to participate in such educational programs only through communication, encouragement and education.

### Effect of the education on the use of systematic reviews in clinical practice

All 12 participants stated that this education would encourage them to use SRs to address clinical issues in their clinical practice. Seven participants indicated a willingness to use SRs to seek answers to clinical questions encountered in medical practice with the goal of improving patient care. Two stated that they use SRs to educate students in their practice as teachers. One participant, however, stated that they were not sure whether they could independently implement the knowledge from SRs in their clinical practice.

I1: „…*so it’s an eternal question for me. I can open a systematic review at home when I have a doubt. But in my daily work, I think that guidelines should be made from systematic reviews, I don’t know, I don’t think that I, alone, could take a systematic review and, according to its results, make some clinical decisions in my work, and a colleague of mine could make different decisions. That part of the use of systematic reviews that somehow remains unclear to me*.“.

One participant expressed a strong opinion of the need to use SRs and scientific evidence in general in medical practice:

I12: „…*people need to understand that, most of the actions, like in nursing, there are a lot of actions that are written based on someone’s opinion and not based on something relevant, and then, based on that opinion, guidelines are created. People just need to be educated that evidence should be a number one source of information on which we should base clinical action, especially in 2021, as we have such extraordinary information the procedures could be based really on evidence.“*.

## Discussion

In this study, we conducted pilot-testing among HCWs of our new short online educational intervention about SRs. HCWs provided positive feedback about the education, suggestions that could improve the educational intervention and ideas on how HCWs could be motivated to take part in such an education. Based on these findings, educational intervention can be improved and further tested on a larger sample of participants.

In this study, we used purposeful sampling to find the targeted participants. Purposeful sampling as a technique is widely used in qualitative research for the identification and selection of information-rich cases for the most effective use of limited resources. This involves identifying and selecting individuals or groups of individuals that are especially knowledgeable about or experienced with a phenomenon of interest. In addition to knowledge and experience, availability and willingness to participate, and the ability to communicate experiences and opinions in an articulate, expressive, and reflective manner are very important [20].

Due to the lack of similar studies, we cannot compare our results with other studies focused on examining knowledge about SRs among HCW. On a related topic, Munroe et al. assessed self-reported knowledge of nurses about EBP in which only 3% of nurses declared that they were very familiar with EBP [21]. While it is acknowledged that knowledge of EBP is a much wider concept than knowledge of SRs only, the results of that study are discouraging, considering the importance of SRs in the hierarchy of evidence.

Although medical education has a tradition of a pedagogical approach to learning based on face-to-face teaching through a teacher-centered model [22], online models of HCW education have been proven successful [23]. Most of our participants stated that they find educational materials like these appropriate for learning about SRs.

Most participants declared that the online format is appropriate for learning about SRs. The advantage of online learning during the pandemic was also highlighted. E-learning came into considerable focus during the current COVID-19 pandemic, in which numerous universities, including ones providing healthcare education, switched entirely to e-learning [24]. Even during the pandemics, HCWs must have accessible education, which might necessitate the transition from face-to-face learning to online learning in times of social distancing. Further studies about online education are, thus, welcome.

In our study, most participants mentioned the conciseness of the text as an advantage. It is known that the amount of text and reader-friendly content affects the acceptance of educational materials [25]. Based on the feedback of the participants, our educational intervention was adequate in this respect.

Furthermore, all participants agreed that the duration of our online education was adequate. With the online platform we used, we could not measure separately how much time the participants spent in reading the educational modules we prepared, and how much time they needed to fill out the questionnaires. Thus, it is worth emphasizing that the duration of educational intervention only, without questionnaires, is shorter than half an hour on average. Most participants stated that the optimal duration of online education could be 30–45 min, which compares to the classical duration of one school class. We planned our intervention purposefully to be brief, due to the studies indicating that the most common obstacle among HCWs for practicing evidence-based medicine (EBM) was lack of time [5, 26]. Thus, long intervention could be rejected a priori by HCWs, and knowing that the education would not take much of their time could motivate them to participate.

All participants declared that, after this education, they would have more confidence to seek answers to clinical questions in SRs of literature. Three-quarters of participants expressed willingness to use knowledge from SRs in their clinical or teaching practice. These findings confirm the association between EBM knowledge and willingness to use evidence gathered from the scientific literature in clinical practice [27]. Nevertheless, one participant expressed doubt about altering the established clinical practice based on knowledge gained from SRs or scientific literature in general. This shows that although HCWs may have confidence in SRs and evidence-based scientific literature, they are not certain if they should interpret and apply results themselves.

While the intervention improved the short-term knowledge of participants after the intervention, most of the wrong answers before and after the intervention referred to forest and funnel-plot. Poor self-assessed knowledge of graphical presentations in meta-analysis has been described elsewhere in the literature [7, 28, 29]. Thus, our results are aligned with prior research in this respect.

We did not intend to conduct a full-scale quantitative study. However, our preliminary results of knowledge testing indicated an absolute increase of 20% in the number of correctly answered questions after the intervention. It has been reported elsewhere that educational workshops and similar educational interventions contributed to increasing knowledge of EBP from 3 to 28% [21]. Thus, our results are in line with prior research results.

George et al. conducted an SR about the effectiveness of digital education (online and local area network-based) in improving practicing physicians’ knowledge, skills, attitude, satisfaction, practice or behavior change, patient outcomes, and cost-effectiveness [30]. They included 93 studies (N = 16,895), of which 76 compared digital education (including blended) and self-directed/face-to-face learning. Their findings revealed that digital education and blended learning may be equivalent to self-directed/face-to-face learning for training practicing physicians. However, the quality of the included evidence for knowledge was very low and the authors suggested that new high-quality randomized controlled trials are needed to confirm those findings [30]. Our study adds to the evidence in this field, but it is worth emphasizing that this was a pilot study, warranting further testing of the intervention.

In our study, two-thirds of participants stated after an educational intervention that they would search for an answer to their clinical question in the scientific literature in general, and a quarter said they would search for an answer in an SR, which re-emphasizes knowledge of EBM as a prerequisite for using evidence in clinical practice [27]. These answers were theoretical, and it is unclear whether this new knowledge would change the participants’ behavior in practice.

In 2017, Rohwer et al. published a Campbell SR, which investigated the effectiveness of e-learning in improving knowledge and EBHC. The review included studies of various designs involving any HCW evaluating any kind of educational intervention about EHBC and which was conducted either completely (pure e-learning) or in part (combined learning) via an electronic platform, in comparison to no learning, learning face-to-face or other forms of e-learning about EBHC. The SR included various interventions. The results showed that, compared to no learning, pure e-learning improves the knowledge and skills of EBHC, but not attitudes and behaviors [31]. In our study, we did not explore skills and behavior; thus, this should be explored in future studies of the intervention we created.

It is worth emphasizing that online education can be synchronous or asynchronous. In asynchronous online learning, students can access educational materials at any time they choose. On the contrary, synchronous online learning includes live instruction, requiring participants to participate at a specific time. Our online education about SRs was designed as asynchronous. It is possible that the type of online education, i.e. synchronous versus asynchronous, can influence the education outcomes. In 2020, Nieuwoudt explored different types of online education among university students and reported that it was important for students to attend class, but it did not necessarily make a difference whether students attended synchronous virtual classes or watched the recordings of the virtual classes [32]. The study found no difference in academic success depending on whether students attended synchronous or asynchronous virtual classes [32]. Further studies in this field could explore whether our intervention would yield different results if we would compare synchronous and asynchronous delivery.

Finally, the presented summaries of systematic and non-systematic narrative reviews were, after the educational intervention, accurately identified as such in most cases (67–92%), but neither one abstract was accurately classified by all participants, indicating an additional need for practical application of knowledge about SRs to reading and interpretation of scientific literature.

Multiple studies have employed qualitative methodology to seek opinions about educational interventions from HCWs [33–36]. Such studies enable authors to test the intervention on a smaller sample before analyzing the efficacy of an intervention in the larger, adequately powered quantitative study. This is also our plan. First, we plan to revise the educational intervention based on participants’ suggestions. As an additional explanation of the forest and funnel plot and adding digital content or images to the education were mentioned by several participants, we plan to add a graphical explanation of the forest and funnel plot to the educative materials. Additionally, based on a suggestion received, we will add a description of all steps involved in a production of an SR. Then we plan to further test the efficacy of the modified version for increasing knowledge.

### Strengths and limitations

The strength of this study includes collecting the results of the pre-intervention and post-intervention questionnaire, followed by interviews. Furthermore, quotations from the interviews were presented in the results to convey as accurately as possible the thoughts and attitudes of the participants about this educational intervention, SRs and scientific literature in general.

The limitation of the study may be the choice of participants, who were relatively younger, and thus not necessarily representative of the population of HCW. Younger participants may be more prone to online learning and more familiar with digital technologies. These participants were suggested by their former university teachers; thus, they could have been more successful students, more motivated for education and scientific advancement, and had more initial knowledge of SRs than the average HCW with a similar level of education. Furthermore, the online platform used for this study does not provide information about the time spent reading the intervention. Thus, we do not know how much time it took the participants to read through the intervention or whether they actually read the educational texts. However, considering the observed improvement in knowledge, we believe it is safe to assume that the participants have read the intervention.

Our intervention included passive online teaching without any interaction. Future studies should compare such teaching mode with interactive learning methods and compare their impact on acquired knowledge and translation into practice.

In this study, we evaluated knowledge immediately after participants’ reading of the online module. Future studies should explore knowledge retention after a longer period. Future studies could consider more active learning approaches for the modules instead of simply pure text. Furthermore, a staggered release of the educational text can also be tested, as this could impact knowledge retention.

In our study, we did not aim to test the impact of our intervention on clinical practice. The ultimate goal of medical education is to influence practice and lead to practical changes in the practice, where needed. Future studies should try to measure the impact of such educational interventions on clinical practice.

Our study was focused only on SRs, while it needs to be acknowledged that clinical decisions are based not only on SRs, but on multiple sources of evidence.

Also, it is possible that with less structured questions in the qualitative part of the study, we would get different responses from the participants. However, since we were testing an intervention, we wanted to make sure with our questions that we covered everything we found important about the intervention.

## Conclusion

Online educational intervention on SRs was pilot-tested among HCWs, which enabled us to collect detailed opinions about the intervention and to collect ideas for improving education, which can be used to tailor the education for end-users. In future studies, it would be useful to examine the effectiveness of the modified educational intervention on increasing knowledge in a larger sample and in the form of a randomized controlled trial.

## Electronic supplementary material

Below is the link to the electronic supplementary material.

Supplementary Material 1

Supplementary Material 2

Supplementary Material 3

## Data Availability

Raw data generated in the pre-intervention and post-intervention questionnaires are available in Supplementary file 8. Interviews were conducted in Croatian. Interview transcripts can be obtained from the corresponding author on request.

## References

[1] Ngim CF, Ibrahim H, Abdullah N, Lai NM, Tan RKM, Ng CS, Ramadas A (2019). A web-based educational intervention module to improve knowledge and attitudes towards thalassaemia prevention in Malaysian young adults. Med J Malay.

[2] Vinokur AD, Merion RM, Couper MP, Jones EG, Dong Y (2006). Educational web-based intervention for high school students to increase knowledge and promote positive attitudes toward organ donation. Health Educ Behav.

[3] Rahimi-Ardabili H, Spooner C, Harris MF, Magin P, Tam CWM, Liaw ST, Zwar N (2021). Online training in evidence-based medicine and research methods for GP registrars: a mixed-methods evaluation of engagement and impact. BMC Med Educ.

[4] Puljak L: The difference between evidence-based medicine, evidence-based (clinical) practice and evidence-based health care. J Clin Epidemiol 2021, In press. doi: https://doi.org/10.1016/j.jclinepi.2021.11.015. 10.1016/j.jclinepi.2021.11.015 34780980

[5] Barzkar F, Baradaran HR, Koohpayehzadeh J (2018). Knowledge, attitudes and practice of physicians toward evidence-based medicine: A systematic review. J Evid Based Med.

[6] Hong J, Chen J. Clinical Physicians’ Attitudes towards Evidence-Based Medicine (EBM) and Their Evidence-Based Practice (EBP) in Wuhan, China. Int J Environ Res Public Health 2019, 16(19). 10.3390/ijerph16193758 PMC6801589 31591286

[7] Puljak L, Sapunar D: Acceptance of a systematic review as a thesis: survey of biomedical doctoral programs in Europe. Syst Rev 2017, 6(1):253. 10.1186/s13643-017-0653-x PMC5727923 29233170

[8] Varnell G, Haas B, Duke G, Hudson K (2008). Effect of an educational intervention on attitudes toward and implementation of evidence-based practice. Worldviews Evid Based Nurs.

[9] Stevenson K, Lewis M, Hay E (2004). Do physiotherapists’ attitudes towards evidence-based practice change as a result of an evidence-based educational programme?. J Eval Clin Pract.

[10] Shuval K, Berkovits E, Netzer D, Hekselman I, Linn S, Brezis M, Reis S (2007). Evaluating the impact of an evidence-based medicine educational intervention on primary care doctors’ attitudes, knowledge and clinical behaviour: a controlled trial and before and after study. J Eval Clin Pract.

[11] Tong A, Sainsbury P, Craig J (2007). Consolidated criteria for reporting qualitative research (COREQ): a 32-item checklist for interviews and focus groups. Int J Qual Health Care.

[12] Cochrane interactive learning: conducting an intervention review. Available at: https://training.cochrane.org/interactivelearning.

[13] Krnic Martinic M, Pieper D, Glatt A, Puljak L (2019). Definition of a systematic review used in overviews of systematic reviews, meta-epidemiological studies and textbooks. BMC Med Res Methodol.

[14] Sharma R, Lakhani R, Rimmer J, Hopkins C. Surgical interventions for chronic rhinosinusitis with nasal polyps. Cochrane Database of Systematic Reviews 2014(11). 10.1002/14651858.CD006990.pub2 PMC11166467 25410644

[15] Lissiman E, Bhasale AL, Cohen M. Garlic for the common cold. Cochrane Database of Systematic Reviews 2014(11). 10.1002/14651858.CD006206.pub4 PMC6465033 25386977

[16] Bogduk N, Dreyfuss P, Govind J (2009). A narrative review of lumbar medial branch neurotomy for the treatment of back pain. Pain Med.

[17] Lam SKK, Kwong EWY, Hung MSY, Pang SMC, Chiang VCL (2018). Nurses’ preparedness for infectious disease outbreaks: A literature review and narrative synthesis of qualitative evidence. J Clin Nurs.

[18] Krnic Martinic M, Meerpohl JJ, von Elm E, Herrle F, Marusic A, Puljak L (2019). Attitudes of editors of core clinical journals about whether systematic reviews are original research: a mixed-methods study. BMJ Open.

[19] Neergaard MA, Olesen F, Andersen RS, Sondergaard J (2009). Qualitative description - the poor cousin of health research?. BMC Med Res Methodol.

[20] Palinkas LA, Horwitz SM, Green CA, Wisdom JP, Duan N, Hoagwood K (2015). Purposeful Sampling for Qualitative Data Collection and Analysis in Mixed Method Implementation Research. Adm Policy Ment Health.

[21] Munroe D, Duffy P, Fisher C (2008). Nurse knowledge, skills, and attitudes related to evidence-based practice: before and after organizational supports. Medsurg Nurs.

[22] O’Doherty D, Dromey M, Lougheed J, Hannigan A, Last J, McGrath D (2018). Barriers and solutions to online learning in medical education - an integrative review. BMC Med Educ.

[23] Lei T, Yu X, Zou M, Wang P, Yuan RH (2021). Delivering an online course in emergency nursing education during the pandemic: What are the effects on students’ learning?. Australas Emerg Care.

[24] Puljak L, Čivljak M, Haramina A, Mališa S, Čavić D, Klinec D, Aranza D, Mesarić J, Skitarelić N, Zoranić S (2020). Attitudes and concerns of undergraduate university health sciences students in Croatia regarding complete switch to e-learning during COVID-19 pandemic: a survey. BMC Med Educ.

[25] Mills R, Haga SB (2018). Qualitative user evaluation of a revised pharmacogenetic educational toolkit. Pharmgenomics Pers Med.

[26] Nejašmić D, Vrdoljak D, Bralić Lang V, Borovac JA, Marušić A (2020). Awareness, attitudes, barriers, and knowledge about evidence-based medicine among family physicians in Croatia: a cross-sectional study. BMC Fam Pract.

[27] Li S, Cao M, Zhu X (2019). Evidence-based practice: Knowledge, attitudes, implementation, facilitators, and barriers among community nurses-systematic review. Med (Baltim).

[28] Badenes-Ribera L, Frias-Navarro D, Iotti NO, Bonilla-Campos A, Longobardi C (2018). Perceived Statistical Knowledge Level and Self-Reported Statistical Practice Among Academic Psychologists. Front Psychol.

[29] Badenes-Ribera L, Frias-Navarro D, Pascual-Soler M, Monterde IBH (2016). Knowledge level of effect size statistics, confidence intervals and meta-analysis in Spanish academic psychologists. Psicothema.

[30] George PP, Zhabenko O, Kyaw BM, Antoniou P, Posadzki P, Saxena N, Semwal M, Tudor Car L, Zary N, Lockwood C (2019). Online Digital Education for Postregistration Training of Medical Doctors: Systematic Review by the Digital Health Education Collaboration. J Med Internet Res.

[31] Rohwer A, Motaze NV, Rehfuess E, Young T (2017). E-learning of evidence-based health care (EBHC) to increase EBHC competencies in healthcare professionals: a systematic review. Campbell Syst Reviews.

[32] Nieuwoudt JE (2020). Investigating synchronous and asynchronous class attendance as predictors of academic success in online education. Australasian J Educational Technol.

[33] Young B, Fogarty AW, Skelly R, Shaw D, Sturrock N, Norwood M, Thurley P, Lewis S, Langley T, Cranwell J (2020). Hospital doctors’ attitudes to brief educational messages that aim to modify diagnostic test requests: a qualitative study. BMC Med Inform Decis Mak.

[34] Pallesen KS, Rogers L, Anjara S, De Brún A, McAuliffe E (2020). A qualitative evaluation of participants’ experiences of using co-design to develop a collective leadership educational intervention for health-care teams. Health Expect.

[35] Kesten JM, Davies CF, Gompels M, Crofts M, Billing A, May MT, Horwood J (2019). Qualitative evaluation of a pilot educational intervention to increase primary care HIV-testing. BMC Fam Pract.

[36] Ha L, Pepin J (2018). Clinical nursing leadership educational intervention for first-year nursing students: A qualitative evaluation. Nurse Educ Pract.

