# Supplementary material for: Creating an online educational intervention to improve knowledge about systematic reviews among healthcare workers: mixed-methods pilot study
Source: BMC Med Educ. 2022 Oct 14;22:722. doi: 10.1186/s12909-022-03763-3 (PMC9562058; doi:10.1186/s12909-022-03763-3)
Supplement: Supplementary file 1 — Supplementary Material 1 [file 12909_2022_3763_MOESM1_ESM.docx]

**List of supplementary files**

**Supplementary file 1.** Invitation to participate in a qualitative study about an educational intervention – a semi-structured interview

**Supplementary file 2.** The text of the pre-intervention and post-intervention questionnaires

**Supplementary file 3.** Educational intervention

**Supplementary file 4.** Four selected article abstracts for assessment

**Supplementary file 5.** The text of the informed consent document

**Supplementary file 6.** Questions for the semi-structured interview

**Supplementary file 7.** Results of the pre-intervention and post-intervention questionnaires

**Supplementary file 8.** Raw data collected within the study via the pre-intervention and post-intervention questionnaires (Excel file)
