# Supplementary material for: Creating an online educational intervention to improve knowledge about systematic reviews among healthcare workers: mixed-methods pilot study
Source: BMC Med Educ. 2022 Oct 14;22:722. doi: 10.1186/s12909-022-03763-3 (PMC9562058; doi:10.1186/s12909-022-03763-3)
Supplement: Supplementary file 2 — Supplementary Material 2 [file 12909_2022_3763_MOESM2_ESM.docx]

**Supplementary file 1. Invitation to participate in a qualitative study about an educational intervention – a semi-structured interview**

E-mail subject: Education about systematic reviews: invitation to participate in a study

Dear colleagues,

Hereby we invite you to participate in education about systematic reviews. In this study, we are interested in your feedback regarding such education. During this study, you will first access online education about systematic reviews. The education will be carried out online. After going through the education and completing accompanying questionnaires, you will have a short online interview with a researcher. The expected duration of your participation in the study is 30 minutes.

The interviews will be conducted one-on-one via videoconferencing software such as Skype, Zoom or MS Teams, according to your preferences and at a time that will be most convenient to you. In the interview, you will be asked pre-defined questions about systematic reviews and the educational intervention you participated in. For every interview, an audio recording will be made, which will be analyzed afterwards. All records will be kept on a secure server. Your participation in this study will help us design better and more useful educational interventions about systematic reviews in the future. After you finish the education, at the end of the online interface, if you wish, we will send you a certificate of participation in the education prepared by Cochrane Croatia.

To access the education and accompanying questionnaires, please follow the link below:

Link XYXYXYXY

Please, answer all questions spontaneously and sincerely.

The study protocol has been approved by the Ethics Committee of the Catholic University of Croatia. There are no risks associated with this study. The level of inconvenience in this study is not higher compared to your experiences in everyday life.

Participation in the study is anonymous. Collected data will be used exclusively for this study and will be saved in a digital form on the computer of the study administrator, protected by a password.

Data access will be provided only for the investigators. Only anonymized results will be used and published for the purpose of improving the quality of education, writing research manuscript and teaching.

Participation in the study is voluntary and is based on your consent. You have the right to withdraw from the study at any time, without any consequences

According to applicable law, you have the right to access your personal data, correction, deletion, restriction of analysis and portability of personal data and the right to object against processing and filing a complaint to the Croatian Personal Data Protection Agency.

If, after completion of the study, you would like to be informed about its results and conclusions or you have questions or claims regarding the study, please contact the principal investigator via an e-mail (prof. dr. sc. Livia Puljak, livia.puljak@unicath.hr).

If you have any complaints about the procedure or you have any concerns about something you have experienced during your participation in the study, please, contact the principal investigator of this study (prof. dr. sc. Livia Puljak, livia.puljak@unicath.hr).

We sincerely ask you to accept our invitation and participate in the study.

Best regards,

Marina Krnić Martinić, MD

**Supplementary file 2. The text of the pre-intervention and post-intervention questionnaires**

First page of SurveyMonkey platform: an introductory statement

Dear Colleagues,

Thank you for participating in the study. By following the link, you are entering the interface for conducting the education, confirming that you are participating in the study voluntarily and that you are giving informed consent for participation.

**Pre-intervention questionnaire**

1. Select your University Department of Health Studies:

1. Catholic University of Croatia

2. University Department of Health Studies Split

3. University Department of Health Studies Zadar

4. University of Dubrovnik, Nursing Studies

5. University North

6. Faculty of Dental Medicine and Health, University of Osijek

7. Faculty of Health Studies, University of Rijeka

8. Medical School, University of Zagreb

2. Your field of studies:

Nursing

Physiotherapy

Radiological technologies

Medical laboratory diagnostics

Midwifery

3. Your study year:

1

2

4. Are you currently employed?

Yes

No

5. Are you currently employed as a healthcare worker?

Yes

No

5a. If yes, how many years of experience working as a healthcare worker do you have? _______ (years)

6. How old are you: ___________years

7. Your sex:

Male

Female

I do not wish to declare

8. How would you rate your knowledge about evidence-based medicine and scientific methodology? Use grades from 1 (insufficient knowledge) to 5 (excellent knowledge).

1 2 3 4 5

9. Have you ever heard of a type of study/scientific article called systematic review?

Yes

No

If your answer to question #9 was yes:

10. Where have you heard about systematic reviews: ______________ (enter an answer)

11. Have you ever read a systematic review?

Yes

No

Other answer: _______________

12. Have you ever participated in producing a systematic review?

Yes

No

Please assess the following statements:

13. In a systematic review, it is enough to search one database

Yes

No

I am not sure

I do not know

14. A true systematic review can be done by a single author

Yes

No

I am not sure

I do not know

15. Systematic reviews must contain a meta-analysis

Yes

No

I am not sure

I do not know

16. While producing a systematic review, two authors should independently assess studies found in the literature search and should independently extract data from the included studies.

Yes

No

I am not sure

I do not know

17. A systematic review should provide a list of included and excluded studies.

Yes

No

I am not sure

I do not know

18. A systematic review should contain a quality assessment of the included studies.

Yes

No

I am not sure

I do not know

19. If a statistical analysis called meta-analysis is performed, an assessment of heterogeneity (diversity) should be performed to verify if the studies are comparable.

Yes

No

I am not sure

I do not know

20. Meta-analysis results are displayed as a graph called funnel plot:

Yes

No

I am not sure

I do not know

21. Publication bias assessment results are displayed as a graph called forest plot.

Yes

No

I am not sure

I do not know

22. Read the following characteristics and state if you agree whether a systematic review should contain those characteristics. Express your agreement with a number on a scale from 1 to 5 that best suits your opinion, where 1 means "completely disagree" and 5 "Completely agree".

i) A research question has been defined 1 2 3 4 5

ii) Listed sources of literature searched, with repeatable search strategy (naming of databases, naming of search platforms, search date and complete search strategy) 1 2 3 4 5

iii) Listed criteria for inclusion and exclusion of research 1 2 3 4 5

iv) Listed selection methods 1 2 3 4 5

v) Critically evaluates and reports on the quality/risk of bias of the included studies 1 2 3 4 5

vi) Provides information on data analysis and synthesis that allows repeatability of results 1 2 3 4 5

**Post -intervention questionnaire**

After reading the information shown, please answer the following questions:

Please assess the following statements:

13. In a systematic review, it is enough to search one database

Yes

No

I am not sure

I do not know

14. A true systematic review can be done by a single author

Yes

No

I am not sure

I do not know

15. Systematic reviews must contain a meta-analysis

Yes

No

I am not sure

I do not know

16. While producing a systematic review, two authors should independently assess studies found in the literature search and should independently extract data from the included studies.

Yes

No

I am not sure

I do not know

17. A systematic review should provide a list of included and excluded studies.

Yes

No

I am not sure

I do not know

18. A systematic review should contain a quality assessment of the included studies.

Yes

No

I am not sure

I do not know

19. If a statistical analysis called meta-analysis is performed, an assessment of heterogeneity (diversity) should be performed to verify if the studies are comparable.

Yes

No

I am not sure

I do not know

20. Meta-analysis results are displayed as a graph called funnel plot:

Yes

No

I am not sure

I do not know

21. Publication bias assessment results are displayed as a graph called forest plot.

Yes

No

I am not sure

I do not know

22. Read the following characteristics and state if you agree whether a systematic review should contain those characteristics. Express your agreement with a number on a scale from 1 to 5 that best suits your opinion, where 1 means "completely disagree" and 5 "Completely agree".

i) A research question has been defined 1 2 3 4 5

ii) Listed sources of literature searched, with repeatable search strategy (naming of databases, naming of search platforms, search date and complete search strategy) 1 2 3 4 5

iii) Listed criteria for inclusion and exclusion of research 1 2 3 4 5

iv) Listed selection methods 1 2 3 4 5

v) Critically evaluates and reports on the quality/risk of bias of the included studies 1 2 3 4 5

vi) Provides information on data analysis and synthesis that allows repeatability of results 1 2 3 4 5

11. How would you use systematic reviews in your clinical practice? ______________ (please state)

12. If you would need to look for information to solve a clinical problem, where would you look for that information? (multiple answers are possible)

a) From colleagues at work

b) In books

c) In scientific literature

d) In a systematic review

e) On an Internet search engine such as Google

f) Somewhere else: _________________________ (please state)

Now please evaluate the four abstracts from the literature. For each abstract, evaluate whether it describes a systematic review of the literature or not.

7. Abstract 1. Is this an abstract of a systematic review of the literature? Yes / No

8. Abstract 2. Is this an abstract of a systematic review of the literature? Yes / No

9. Abstract 3. Is this an abstract of a systematic review of the literature? Yes / No

10. Abstract 4. Is this an abstract of a systematic review of the literature? Yes / No

If you want to get a certificate for attending this training, please leave your name and e-mail address. We will not use your name and e-mail address for any purpose other than sending the certificate; after completing the study, we will delete your name and e-mail address from the data collected.

Name and surname:

E-mail address:

Thank you for participating in the education and research.

**Supplementary file 3. Educational intervention**

Please, now read the 11 short modules with information on systematic reviews:

*1. Definition of evidence-based medicine*

Evidence-based medicine is an approach to health care that combines the experience of health professionals with the wishes and needs of patients and the best scientific evidence.

*2. Hierarchy of evidence in medicine*

Not all evidence in medicine is considered equally valuable. The hierarchy of evidence in medicine is depicted as a pyramid; at the top of the pyramid, there is evidence we can trust more (evidence which is more reliable and with a lower risk of bias), and at the bottom is evidence that we can trust less (less reliable, with a higher risk of bias).

At the top of this pyramid are randomized clinical trials conducted on humans and systematic reviews of the literature that combine the results of individual studies. In the middle of the pyramid are perceptual research, and at the bottom of the pyramid are personal opinions, research on animals, cells, and models.

*3. Systematic review of the literature*

A systematic review of the literature is a type of research that combines the results of research conducted on a specific, pre-defined topic. Such a review of the literature is called “systematic” because there is a demanding methodology that needs to be followed in order for a systematic review to be properly conducted. Such research includes asking a clinical question, systematically searching the literature, analyzing data, and drawing conclusions based on all research available on a particular topic. All these criteria are agreed upon and defined in advance, before the start of the study.

While conducting a systematic review, multiple scientific research databases should be searched to increase the likelihood of finding all studies available on a defined topic.

A series of steps during the conduct of a systematic review should be done by two authors independently, after which the results of their work are compared. This is done to avoid accidental errors and bias.

The six key features of a systematic review are:

1. A research question is defined

2. Listed literature sources searched, with repeatable search strategy (naming of databases, naming of search platforms, search dates and complete search strategy)

3. Listed criteria for inclusion and exclusion of research

4. Listed selection (screening) methods

5. Critically evaluates and reports on the quality/risk of bias of the studies included

6. Provides information on the analysis and synthesis of data that allow repeatability of results.

*4. Beginning of a systematic review production: asking a clinical question*

A systematic review of the literature begins with asking the research question, which will be investigated. The research question usually contains the so-called PICO elements (Patient/health problem, Intervention, Comparison/control, Outcome), i.e. a description of the patients’ characteristics, the intervention to be investigated, the control with which the intervention will be compared (comparator) and the planned outcomes.

An example of a research question for a systematic review of the literature is:

Can a single dose of ibuprofen 400 mg relieve postoperative pain in adults after wisdom tooth extraction, compared to placebo?

In this research question, patients are adults who need a wisdom tooth extraction; the study intervention is 400 mg of ibuprofen in a single dose, the comparison is placebo, and the planned outcome is postoperative pain.

*5. Writing a systematic review protocol and registering the protocol*

When defining a research question, it is necessary first to write a systematic review protocol, which will describe in detail the planned methods, including:

- indication of inclusion and exclusion criteria (types of research to be included, characteristics of respondents, types of comparisons, types of outcomes)

- naming the databases to be searched and by which search strategy,

- research selection methods that meet the inclusion criteria,

- the way in which the data from the included surveys will be extracted,

- a description of the methods of critical assessment of quality, i.e. the risk of bias in the included research

- method of assessing the credibility of evidence,

- information on data analysis and synthesis (statistical analysis, meta-analysis)

- method of handling missing data,

- method of assessing the heterogeneity (diversity) of the included research,

- analysis of publishing bias,

- sensitivity analysis.

It is desirable to publish or register the protocol in a publicly available register on the Internet so that the planned methods of a systematic review are publicly available and transparent.

*6. Literature search*

In a systematic review of the literature, it is necessary to search at least two electronic databases of scientific research, e.g. MEDLINE or PubMed, and Embase. Searching a larger number of scientific databases increases the probability of finding a larger number of papers on a given topic. Some databases of scientific papers are specialized and are used only for certain topics. The search of electronic databases should be Supplementary fileed by searching additional sources of literature, such as registers of clinical trials, dissertations, unpublished research, conference abstracts, and references and citations of included research.

Complex search strategies are used to search the literature, including the use of pre-defined medical terms and free words in the text. An information specialist should be involved in developing a complex search strategy.

*7. Screening the literature*

After searching the literature, at least two authors should first independently review all titles and abstracts obtained by searching and exclude those that certainly do not meet the inclusion criteria. The full texts of the records retained in the first phase as eligible or potentially eligible are analyzed in the next phase. After full-text analysis, only studies that meet the eligibility criteria are included. The duration of literature screening obtained by searching can be very long, depending on the complexity of the topic. Literature search can yield thousands or tens of thousands of records that need to be screened.

*9. Research quality and risk of bias*

The quality of all studies included in the systematic review needs to be assessed to determine the potential risk of bias. Namely, not all research is done equally well, and poor research is not reliable. Therefore, to adequately assess the reliability of the data collected by a systematic review, it is necessary to know whether we can trust the included studies. The risk of bias assessment includes an analysis of multiple methodological features of the included studies, from randomization into groups, concealment of randomization, concealment of the obtained intervention and measurement of outcomes, respondents’ attrition, selective reporting and any other source of bias. The risk of bias assessment tells us whether the methods were inappropriate or unclear and whether they could have affected the reliability of the data. Standardized instruments are used for this assessment.

*10. Data analysis*

If the studies included in a systematic review are sufficiently similar, a statistical analysis of the data called meta-analysis can be used. In a meta-analysis, the results collected from several studies are analyzed together. A meta-analysis, therefore, allows us to aggregate the results of a larger number of studies numerically and thus obtain more reliable evidence than any of these studies separately. The results of the meta-analysis are presented in a graph called a forest plot.

Data from meta-analyses can be used to assess the risk of publishing bias, which is presented by a graph called a funnel plot. Such a graph shows whether the results from individual studies were mostly positive or negative for the examined intervention, which could mean that studies that gave different results were intentionally not published.

*11. Interpretation of data - conclusion for clinical practice and recommendations for future research*

After analyzing the data, the data is interpreted, and conclusions are drawn for future clinical practice and recommendations made for future research. While making conclusions, the risk of bias assessment and certainty of the evidence for specific outcomes should be taken into consideration. If the included studies are not of high quality and reliable, then it is important to conclude that we cannot fully trust them and that new research on this topic is necessary.

A systematic review clearly and accurately displays all available studies on a particular topic, from multiple databases and with reduced researcher bias; puts the results in context by comparing different researches, and helps to determine which new researches are needed by outlining what has been done, how well and with what results.

**Supplementary file 4. Four selected article abstracts for assessment**

**Abstract 1**

**Surgical interventions for chronic rhinosinusitis with nasal polyps** **[1]**

**Abstract**

**Background:**Surgical treatment of chronic rhinosinusitis with nasal polyps is an established treatment for medically resistant nasal polyp disease. Whether a nasal polypectomy with additional sinus dissection offers any advantage over an isolated nasal polypectomy has not been systematically reviewed.

**Objectives:**To assess the effectiveness of simple polyp surgery versus more extensive surgical clearance in chronic rhinosinusitis with nasal polyps.

**Search methods:**We searched the Cochrane Ear, Nose and Throat Disorders Group Trials Register; the Cochrane Central Register of Controlled Trials (CENTRAL 2014, Issue 1); PubMed; EMBASE; CINAHL; Web of Science; Cambridge Scientific Abstracts; ICTRP and additional sources for published and unpublished trials. The date of the search was 20 February 2014.

**Selection criteria:**Randomized and quasi-randomized controlled trials in patients over 16 with chronic rhinosinusitis with nasal polyps, who have failed a course of medical management and who have not previously undergone any previous surgical intervention for their nasal disease. Studies compared nasal polypectomy with more extensive sinus clearance in this patient cohort.

**Data collection and analysis:**We used the standard methodological procedures expected by The Cochrane Collaboration.

**Main results:**We identified no trials which met our inclusion criteria. Six controlled trials (five randomized) met some but not all of the inclusion criteria and were therefore excluded from the review.

**Authors' conclusions:**We are unable to reach any conclusions as to whether isolated nasal polypectomy or more extensive sinus surgery is a superior surgical treatment modality for chronic rhinosinusitis with nasal polyps. There is a need for high-quality randomized controlled trials to assess whether additional sinus surgery confers any benefit when compared to nasal polypectomy performed in isolation.

**Abstract 2**

**Garlic for the common cold** **[2]**

**Abstract**

**Background:**Garlic is alleged to have antimicrobial and antiviral properties that relieve the common cold, among other beneficial effects. There is widespread usage of garlic supplements. The common cold is associated with significant morbidity and economic consequences. On average, children have six to eight colds per year and adults have two to four.

**Objectives:**To determine whether garlic (allium sativum) is effective for either the prevention or treatment of the common cold, when compared to placebo, no treatment or other treatments.

**Search methods:**We searched the Cochrane Central Register of Controlled Trials (CENTRAL) (2011, Issue 4), which includes the Cochrane Acute Respiratory Infections Group Specialized Register, OLDMEDLINE (1950 to 1965), MEDLINE (January 1966 to November week 3, 2011), EMBASE (1974 to December 2011) and AMED (1985 to December 2011).

**Selection criteria:**Randomized controlled trials of common cold prevention and treatment comparing garlic with placebo, no treatment or standard treatment.

**Data collection and analysis:**Two review authors independently reviewed and selected trials from searches, assessed and rated study quality and extracted relevant data.

**Main results:**Of the six trials identified as potentially relevant from our searches, only one trial met the inclusion criteria. This trial randomly assigned 146 participants to either a garlic supplement (with 180 mg of allicin content) or a placebo (once daily) for 12 weeks. The trial reported 24 occurrences of the common cold in the garlic intervention group compared with 65 in the placebo group (P < 0.001), resulting in fewer days of illness in the garlic group compared with the placebo group (111 versus 366). The number of days to recovery from an occurrence of the common cold was similar in both groups (4.63 versus 5.63). Only one trial met the inclusion criteria, therefore limited conclusions can be drawn. The trial relied on self-reported episodes of the common cold but was of reasonable quality in terms of randomization and allocation concealment. Adverse effects included rash and odor.

**Authors' conclusions:**There is insufficient clinical trial evidence regarding the effects of garlic in preventing or treating the common cold. A single trial suggested that garlic may prevent occurrences of the common cold, but more studies are needed to validate this finding. Claims of effectiveness appear to rely largely on poor-quality evidence.

**Abstract 3**

**Lumbar medial branch neurotomy for the treatment of back pain** **[3]**

**Abstract**

**Background:**Confusion persists concerning the nature and efficacy of procedures variously known as facet denervation, lumbar medial branch radiofrequency neurotomy, and radiofrequency neurotomy or denervation for the treatment of back pain. Systematic reviews have not recognized the importance of patient selection and correct surgical technique when appraising the literature. As a result, negative conclusions about procedures have been drawn because lack of efficacy of one procedure has been misattributed to other, cognate, but different procedures.

**Objectives:**To demonstrate how the rationale and efficacy of lumbar medial branch neurotomy depends critically on correct selection of patients and use of surgically correct technique.

**Methods:**A review and description of the available evidence, drawn from the personal libraries of the authors and from the bibliographies of systematic reviews.

**Results:**Three studies, commonly accepted as evidence of lack of effectiveness, were not valid tests of lumbar medial branch neurotomy because of errors in selection of patients or errors in surgical technique, or both. Two descriptive studies and three controlled studies that used valid or acceptable techniques consistently showed that lumbar medial branch neurotomy had positive effects on pain and disability. All valid, randomized controlled trials showed medial branch neurotomy to be more effective than sham treatment.

**Discussion:**Negative results have been reported only in studies that selected inappropriate patients or used surgically inaccurate techniques. All valid studies showed positive outcomes that cannot be attributed to placebo. Inappropriate conclusions have been drawn by systematic reviews that misrepresent invalid studies as providing evidence against the efficacy of lumbar medial branch neurotomy.

**Abstract 4**

**Nurses' preparedness for infectious disease outbreaks: A literature review and synthesis of qualitative evidence** **[4]**

**Abstract**

**Aims and objectives:**To explore the core components that constitute nurses' preparedness in an epidemic event.

**Background:**Healthcare service providers have worked to augment efforts to protect the public from the impact of epidemic events. While constituting the major healthcare taskforce, nurses are frequently tasked with fronting the response to an infectious disease outbreak. Given the crucial role of nurses in the management of prevailing epidemics, the constituents of their preparedness in epidemic events should be identified.

**Design:**A systematic search was undertaken to identify eligible studies from the literature. A narrative synthesis approach was employed to extract and synthesize findings of the reviewed qualitative studies.

**Methods:**Seven qualitative studies on nurses' experience and perceptions of epidemic events were examined for scientific quality using the Critical Appraisal Skills Program Qualitative Checklist. Findings of these studies were synthesized adopting a narrative synthesis approach.

**Results:**Three interplaying themes were identified as follows: (i) personal resources, (ii) workplace resources and (iii) situational influences. The findings suggest that an effective epidemic outbreak response would require further effort to reinforce the interplay between individual nurses, healthcare institutions and the governments.

**Conclusions:**The practical interplay among individual nurses, healthcare institutions and the government is crucial in establishing an effective epidemic response. Further research on the understanding of the dynamic process of preparedness development is recommended to set future directions in research.

**Relevance to clinical practice:**This study offers important insights for devising future strategies in enhancing nurses' preparedness and response to an epidemic event. These include recommendations on providing education and training to nurses regarding infectious diseases, fostering institutional assistance and support in an outbreak and revising government policies and planning.

**Keywords:**communicable diseases; disease outbreaks; nurses; preparedness.

**References to abstracts:**

1. Sharma R, Lakhani R, Rimmer J, Hopkins C: **Surgical interventions for chronic rhinosinusitis with nasal polyps**. *Cochrane Database of Systematic Reviews* 2014(11).

2. Lissiman E, Bhasale AL, Cohen M: **Garlic for the common cold**. *Cochrane Database of Systematic Reviews* 2014(11).

3. Bogduk N, Dreyfuss P, Govind J: **A narrative review of lumbar medial branch neurotomy for the treatment of back pain**. *Pain Med* 2009, **10**(6):1035-1045.

4. Lam SKK, Kwong EWY, Hung MSY, Pang SMC, Chiang VCL: **Nurses' preparedness for infectious disease outbreaks: A literature review and narrative synthesis of qualitative evidence**. *Journal of clinical nursing* 2018, **27**(7-8):e1244-e1255.

**Supplementary file 5. The text of the informed consent document**

INFORMED CONSENT TO PARTICIPATE IN THE RESEARCH

*Title of the study*: Qualitative evaluation of a pilot online educational intervention to improve knowledge about systematic reviews among healthcare professionals

This study is conducted within a doctoral dissertation of Marina Krnić Martinić, MD. The principal investigator is Prof. Livia Puljak, MD, PhD.

*The aim of the study* is to examine how a short online educational intervention about systematic reviews was experienced by healthcare professionals.

*Study description*: The study will help researchers to improve educational material about systematic reviews for healthcare workers. The participants will contribute to the design of the best possible short educational intervention on systematic reviews for students of biomedical studies and health professionals. Recent graduates of university health sciences studies in Croatia will be invited to participate in the study. The introductory part of the interview will highlight the confidentiality of the information and present the planned content of the interviews. By giving informed consent in the online interface, respondents state that they are aware of the research objectives and agree to participate in the study.

How can research data be used and shared?

The conversation during the interview will be audio-recorded, and then the audio recordings will be transcribed and anonymized. Informed consent forms will be stored separately from audio recordings and transcripts of interviews. Audio recordings will be securely stored and encrypted in SharePoint - a network platform compliant with the General Data Protection Regulation (GDPR), for 5 years from the last published study from this research. The data administrator on the SharePoint platform will be Dr. Marina Krnić Martinić. Information from the interview will be analyzed and the results of the research published. Respondents' personal and identifying information will not be published in any case.

Any respondent may at any time request the deletion of personal data from the research record by sending a simple request to the study coordinator, Prof. Livia Puljak (e-mail contact: livia.puljak@unicath.hr). If respondents do not request the withdrawal from the study, we would like to emphasize that published, anonymized research results cannot be removed (e.g., research results are published in a scientific journal). In order to promote free access to scientific information and avoid unnecessary research, anonymous transcribed data from conducted interviews will be available in the OSF register (Open Science Framework, https://osf.io/). Anonymity will be ensured by removing all names as well as other potential identifiers (country, institution information, etc.).

*Violation of data confidentiality*

In the event of a data breach, we will contact the affected respondents, and the data will be temporarily removed from the compromised storage. Any internal transmission of sensitive data will be done in a secure way. Specifically, secure SharePoint space for this research will be used for data transfer.

*Supervision*

If you want to learn more about data protection methods in this research and at the Catholic University of Croatia, please contact the principal investigator, Prof. Livia Puljak (livia.puljak@unicath.hr). You can also request all information related to the processing of personal data and the exercise of your rights related to the processing of personal data (including the right to withdraw consent) in writing – please use the address Catholic University of Croatia, Ilica 242, 10000 Zagreb or e-mail: sluzbenik@unicath.hr.

*Informed consent*

Participation in this study is voluntary, and respondents can withdraw from the study at any time without giving reasons for withdrawal. If you want to withdraw from the study, please contact the principal investigator, Prof. Livia Puljak (livia.puljak@unicath.hr).

There will be an informed consent form in the online interface where the education you will go through will be posted, where you will be able to give your consent to agree to all the following statements:

• I have read information about the study. I had the opportunity to ask questions and get satisfactory answers. I had enough time to decide whether to participate in this study.

• I am aware that my participation in the study is voluntary. Also, I know that I can choose not to participate or withdraw from this study at any time. I am not obliged to state the reasons for non-participation or withdrawal from the study.

• I consent to the audio recording of the conversation during the interview.

• I consent to the collection and use of my interview data in accordance with applicable data protection guidelines and regulations (GDPR).

• I consent to the secure storage of my interview data on the SharePoint platform for five years from the last published study from this study.

• I consent to my anonymized and transcribed interview data being publicly available on the OSF scientific platform. I understand that this means that my anonymized data may be used for research purposes other than those described above. I am also aware that this means that my anonymized data may be used in countries outside Europe and that data use regulations may be different from those in the European Union.

• I agree to maintain the confidentiality of the information I discussed during the interview.

• I want to participate in this study.

**Supplementary file 6. Questions for the semi-structured interview**

Introduction: You have just finished our educational intervention about systematic reviews.

Questions:

1. How much time did it take to go through the questionnaires and educational intervention?

2. Has the information about systematic reviews presented in our educational intervention changed your knowledge about systematic reviews?

a. If yes, in what way?

b. If yes, which part of the education most significantly contributed to your understanding of systematic reviews?

3. Do you have any dilemmas about systematic reviews after you have finished our educational intervention? If yes, which?

4. Do you find educational materials such as this an appropriate method of learning about systematic reviews?

5. Is the online format appropriate for conduction this sort of intervention among students and healthcare workers? If yes, how so? If not, how so?

6. Do you find the amount of text in the educational intervention appropriate? If yes, why? If not, why?

7. Do you find that the content of the educational intervention has been written appropriately for the purpose of online education about systematic reviews? If yes, why? If not, why?

8. Do you find the duration of the online education appropriate? If yes, why? If not, why?

9. What would be the optimal duration of online education?

10. What would be your suggestions to improve this education about systematic reviews for healthcare workers?

11. How could we encourage healthcare workers to participate in such online educational programs?

12. Would this education encourage you to use systematic reviews to find an answer to a clinical question that might emerge in your clinical practice? If yes, why? If not, why?

Thank you for participating in the study.

**Supplementary file 7. Results of the pre-intervention and post-intervention questionnaires**

***Pre- and post-intervention knowledge assessment***

The results of the knowledge test on the pre-intervention questionnaire are shown in Supplementary table 7.1.

**Supplementary table 7.1. Answers to questions assessing knowledge in the pre-intervention questionnaire (gray shadows indicate wrong answers)**

| Statement | Participant code | | | | | | | | | | | |
| --- | --- | --- | --- | --- | --- | --- | --- | --- | --- | --- | --- | --- |
|  | I1 | I2 | I3 | I4 | I5 | I6 | I7 | I8 | I9 | I10 | I11 | I12 |
| 1. In a systematic review, it is sufficient to search one database | no | no | no | not sure | no | no | no | no | no | no | no | no |
| 2. A true systematic review of the literature can be conducted by only one author | no | not sure | no | not sure | no | no | no | no | no | no | no | not sure |
| 3. Systematic reviews must include a meta-analysis | no | yes | not sure | not sure | not sure | yes | yes | no | no | yes | no | no |
| 4. During the development of systematic reviews, two authors should independently evaluate the research found by searching the literature and independently extract data from the included research. | no | yes | yes | not sure | no | no | not sure | yes | yes | yes | yes | not sure |
| 5. A list of included and excluded research should be provided in the systematic review | yes | not sure | yes | not sure | yes | yes | yes | yes | no | yes | not sure | yes |
| 6. The quality of the research involved should be assessed in a systematic review | yes | not sure | not sure | yes | yes | yes | yes | yes | yes | yes | yes | yes |
| 7. In the case of a statistical analysis called a meta-analysis, an assessment of the heterogeneity (diversity) of the results must be carried out to verify that the studies are comparable. | yes | yes | yes | yes | yes | yes | yes | yes | yes | yes | yes | yes |
| 8. The results of the meta-analysis are presented as a graph called a funnel-plot | no | don’t know | yes | not sure | no | not sure | yes | not sure | yes | not sure | no | yes |
| 9. The results of the publication bias assessment are displayed in a chart called forest-plot | no | don’t know | not sure | yes | not sure | not sure | yes | not sure | no | yes | no | not sure |

None of the participants answered all nine questions assessing knowledge correctly in the pre-intervention questionnaire. Two participants had 8 out of 9 correct answers. The median number of correct answers was 5.5 (IQR 5-7). Overall, of the 108 possible correct answers (12 participants, 9 questions), in the pre-intervention questionnaire, there were 67/108 (62%) questions answered correctly (Supplementary table 7.1). Most of the wrong answers were recorded for the two items about the graphs used in SRs – funnel plot and forest plot (Supplementary table 7.1).

The results of the knowledge test after the intervention are shown in Supplementary table 7.2.

**Supplementary table 7.2. Answers to questions assessing knowledge in the post-intervention questionnaire (gray answers indicate wrong answers)**

| Statement | Participant code | | | | | | | | | | | |
| --- | --- | --- | --- | --- | --- | --- | --- | --- | --- | --- | --- | --- |
|  | I1 | I2 | I3 | I4 | I5 | I6 | I7 | I8 | I9 | I10 | I11 | I12 |
| 1. In a systematic review, it is sufficient to search one database | no | no | no | no | no | no | no | no | no | no | no | no |
| 2. A true systematic review of the literature can be conducted by only one author | no | no | no | no | no | no | no | no | no | no | no | no |
| 3. Systematic reviews must include a meta-analysis | no | yes | yes | yes | yes | yes | yes | no | no | yes | no | no |
| 4. During the development of systematic reviews, two authors should independently evaluate the research found by searching the literature and independently extract data from the included research. | yes | yes | yes | yes | yes | yes | yes | yes | yes | yes | yes | no |
| 5. A list of included and excluded research should be provided in the systematic review | yes | yes | yes | yes | yes | yes | yes | yes | no | yes | yes | no |
| 6. The quality of the research involved should be assessed in a systematic review | yes | yes | yes | yes | yes | yes | yes | yes | yes | yes | yes | Yes |
| 7. In the case of a statistical analysis called a meta-analysis, an assessment of the heterogeneity (diversity) of the results must be carried out to verify that the studies are comparable. | yes | yes | yes | yes | yes | yes | yes | yes | yes | yes | yes | yes |
| 8. The results of the meta-analysis are presented as a graph called a funnel-plot | no | yes | yes | yes | yes | yes | no | no | no | no | no | no |
| 9. The results of the publication bias assessment are displayed in | no | yes | yes | yes | no | no | yes | no | no | yes | no | no |

Four participants answered all questions assessing knowledge about SRs correctly (Supplementary table 7.2). Before the intervention, 24 answers were “not sure” and two answers were “don’t know”; on the contrary, after the intervention, none of the knowledge questions were answered with “not sure” or “don’t know” (Supplementary tables 7.1 and 7.2). The median number of correct answers was 7 (IQR 6.5-9). All participants had more correct answers on the post-intervention knowledge test (Supplementary table 7.2) compared to the pre-intervention (Supplementary table 7.1). Again, the most common wrong answers were related to graphs used in SRs. After the education, 89/108 (82%) items from the post-intervention questionnaire were labelled correctly. Thus, the participants had 20% more correct answers after the intervention than before the intervention.

***Agreement with the proposed characteristics of systematic reviews***

Participants’ agreement with the proposed characteristics of SRs before the intervention are shown in Table 2. Most participants fully agreed with all the claims about SRs; the percentage of complete agreement was 82% (59 participants completely agreed with 72 statements) (Supplementary table 7.3).

**Supplementary table 7.3. Degree of agreement with the statements about systematic reviews presented in the pre-intervention questionnaire (the participants were asked if they agreed that a systematic review should satisfy those characteristics. The degree of agreement was expressed with numbers from 1 to 5, with 1 representing “Completely disagree”, and 5 representing “Completely agree”).**

| Statement | Participant code | | | | | | | | | | | |
| --- | --- | --- | --- | --- | --- | --- | --- | --- | --- | --- | --- | --- |
|  | I1 | I2 | I3 | I4 | I5 | I6 | I7 | I8 | I9 | I10 | I11 | I12 |
| i) A research question is defined | 5 | 5 | 5 | 5 | 4 | 5 | 5 | 5 | 5 | * | 5 | 5 |
| ii) Listed literature sources searched, with repeatable search strategy (naming of databases, naming of search platforms, search dates and complete search strategy) | 5 | 5 | 5 | 5 | 4 | 5 | 5 | 5 | 5 | 5 | 5 | 5 |
| iii) Listed criteria for inclusion and exclusion of research | 5 | 5 | 5 | 5 | 4 | 5 | 5 | 5 | 5 | 5 | 5 | 5 |
| iv) Listed selection (screening) methods | 5 | 4 | 5 | 5 | 4 | 5 | 5 | 5 | 5 | 5 | 5 | 5 |
| v) Critically evaluates and reports on the quality/risk of bias of the studies included | 3 | 4 | 4 | 5 | 4 | 5 | 5 | 5 | 5 | 5 | 5 | 5 |
| vi) Provides information on the analysis and synthesis of data that allow repeatability of results | 5 | 4 | 4 | 5 | 4 | 5 | 5 | 5 | 5 | 5 | 5 | 5 |

*no answer

After the intervention, all participants completely agreed with all 6 proposed SR characteristics in the post-intervention questionnaire, except for the first statement (a research question is defined) on which one participant did not produce an answer (Supplementary table 7.4).

The number of participants who fully agreed with the statements about the characteristics of SRs after the education was 71/72 (99%) (Table 4), representing an increase of 17% compared to the pre-intervention questionnaire (Supplementary table 7.3).

**Supplementary table 7.4. Degree of agreement with the statements about systematic reviews presented in the post-intervention questionnaire (the participants were asked if they agreed that a systematic review should satisfy those characteristics. The degree of agreement was expressed with numbers from 1 to 5, with 1 representing “Completely disagree”, and 5 representing “Completely agree”).**

| Statement | Participant code | | | | | | | | | | | |
| --- | --- | --- | --- | --- | --- | --- | --- | --- | --- | --- | --- | --- |
|  | I1 | I2 | I3 | I4 | I5 | I6 | I7 | I8 | I9 | I10 | I11 | I12 |
| i) A research question is defined | 5 | 5 | 5 | 5 | 5 | 5 | 5 | 5 | 5 | * | 5 | 5 |
| ii) Listed literature sources searched, with repeatable search strategy (naming of databases, naming of search platforms, search dates and complete search strategy) | 5 | 5 | 5 | 5 | 5 | 5 | 5 | 5 | 5 | 5 | 5 | 5 |
| iii) Listed criteria for inclusion and exclusion of research | 5 | 5 | 5 | 5 | 5 | 5 | 5 | 5 | 5 | 5 | 5 | 5 |
| iv) Listed selection (screening) methods | 5 | 5 | 5 | 5 | 5 | 5 | 5 | 5 | 5 | 5 | 5 | 5 |
| v) Critically evaluates and reports on the quality/risk of bias of the studies included | 5 | 5 | 5 | 5 | 5 | 5 | 5 | 5 | 5 | 5 | 5 | 5 |
| vi) Provides information on the analysis and synthesis of data that allow repeatability of results | 5 | 5 | 5 | 5 | 5 | 5 | 5 | 5 | 5 | 5 | 5 | 5 |

*no answer
